# The relationship between fecal incontinence and vaginal delivery in the postmenopausal stage

**DOI:** 10.4274/tjod.56650

**Published:** 2017-03-15

**Authors:** Süleyman Kargın, Sami Çifçi, Adnan Kaynak, Hüseyin Ataseven, Cengiz Kadıyoran, Murat Çakır

**Affiliations:** 1 Medova Private Hospital, Clinic of General Surgery, Konya, Turkey; 2 Karaman State Hospital, Clinic of Gastroenterology, Karaman, Turkey; 3 Necmettin Erbakan University Meram Faculty of Medicine, Department of General Surgery, Konya, Turkey; 4 Necmettin Erbakan University Meram Faculty of Medicine, Department of Gastroenterology, Konya, Turkey; 5 Medova Private Hospital, Clinic of Radiology, Konya, Turkey

**Keywords:** Fecal incontinence, obstetric sphincter injury, postmenopausal stage

## Abstract

**Objective:**

Obstetric anal sphincter injuries are one of the most significant complications of vaginal delivery that give way to fecal incontinence, which is defined as the involuntary leakage of gas, fluid or solid stool. Although sphincter injuries are seen in 0.5-9% of all deliveries. It has been reported that 20-41% of women who had vaginal deliveries had occult anal sphincter injuries as endoanal ultrasonography began to be used by physicians. The aim of our study was to investigate the relationship between fecal incontinence, whose incidence increases dramatically during the postmenopausal stage, and occult anal sphincter injuries.

**Materials and Methods:**

Two hundred healthy female patients with no history of anal sphincter injury, aged between 18 and 70 years were included in the study. The participants were divided into 4 groups according to their menopausal stages and mode of delivery; premenopausal (group 1) and postmenopausal (group 2) vaginal delivery, and premenopausal (group 3) and postmenopausal (group 4) cesarean section. Wexner incontinence scores were determined. The participants’ defects were assessed using endoanal ultrasound and their status of fecal incontinence using anorectal manometric measurements.

**Results:**

Anorectal manometric measurement results were found significantly lower in group 1 than in group 3 (p<0.01). The Wexner scores of groups 1 and 3 were similar. The anorectal manometric measurement results of group 2 were significantly lower than those of group 4, and the Wexner score of group 2 was significantly higher than other groups (p=0.03).

**Conclusion:**

Anal sphincter injuries formed after vaginal delivery may be one of the reasons that increase the incidence of postmenopausal fecal incontinence and cause the formation of fecal incontinence symptoms in women.

## INTRODUCTION

Obstetric anal sphincter injuries (OASIS) account for a significant complication of vaginal deliveries and cause fecal incontinence (FI), which is defined as the involuntary leakage of gas, fluid or solid stool^(1)^. They are seen in 0.5-9% of all deliveries^(2)^. Post-delivery FI incidence, however, is about 3%^(3)^. Only 30% of patients with OASIS were observed to have FI symptoms a year after delivery^(4)^. It has also been reported that rates of fecal emergency and FI in the geriatric population reaches up to 53-80%^(5)^.

As endosonography became more common, occult anal sphincter injuries were detected in most of women with FI. Moreover, it has been shown that 20-41% of women who had normal deliveries but were without FI symptoms had ongoing occult anal sphincter injuries^(6)^. Although there have been ample studies on occult anal sphincter injuries within the last decade, its clinical significance and natural history are still unclear^(7)^. Further, it has also been argued that these injuries might become symptomatic at later ages^(8)^. The reason for the increase seen in FI prevalence in women at later ages is controversial. It has been suggested that the existence of estrogen and progesterone receptors in women led to an increase in FI in the postmenopausal stage because of their hormonal effects on sphincters and pelvic floor muscles^(9)^ but this correlation still proves to be controversial^(10,11)^.

We believe that occult anal sphincter injuries following delivery become symptomatic with changes seen in the postmenopausal stage. Therefore, our aim in this study was to investigate whether occult anal sphincter injuries are among the causes of postmenopausal FIs.

## MATERIALS AND METHODS

Before the study was initiated, the consent of Necmettin Erbakan University, Meram Faculty of Medicine’s Board of Ethics for Clinical Trials was obtained (approval number: 2013/79). Healthy women who presented to the gynecology outpatient clinics of Necmettin Erbakan University Meram Faculty of Medicine between May 2013 and November 2013 with a history of delivery but with no previous history of anal sphincter injury were included in the study. Women aged between 18 and 70 years with at least one delivery and a 6-month interval after their latest delivery were covered by the study. Patients who had been clinically diagnosed as having sphincter injuries and subsequently received treatment for these injuries, and women aged over 70 years were excluded from the study because they were thought to be unsuitable for the tests required. All participants who had vaginal deliveries had a mediolateral episiotomy story at the first birth. Participants who underwent multiple episiotomies or did not have episiotomy were not included in the study. The criteria for inclusion and exclusion in the study are shown in Table 1.

Informed consents were obtained from all participants and they were allocated into 4 groups according to their mode of delivery and menopausal status. Each group had 50 participants, a total of 200 for the whole study. Sample size was set at a minimum of 46 participants for each group with a margin of error of α=0.05 and β=0.20 as revealed by the power analysis performed based on study data presented by Donnelly et al.^(12)^.

### Groups

Group 1: Premenopausal women with a history of vaginal delivery,

Group 2: Postmenopausal women with a history of vaginal delivery,

Group 3: Premenopausal women with a history of c-section (premenopausal control group),

Group 4: Postmenopausal women with a history of c-section (postmenopausal control group).

Premenopausal period: Women aged between 18 and 49 years with at least one delivery and who menstruated at least once in the last 12 months.

Postmenopausal period: Women aged between 49 and 70 years who had no history of menstruation in the last 12 months.

### Evaluation of participants

The age, mode of delivery, number of deliveries, history of postpartum clinical anal sphincter, history of anal sphincter repair, and whether the patients had had symptoms of postpartum FI were questioned. FI scoring was conducted according to the 20-point Wexner incontinence scale (WIS), which is based on patients’ gas, fluid, solid incontinence status and designed to determine changes in lifestyle and the frequency of the need to use pads.

### Anorectal manometric measurement

All subjects received rectum cleansing with a fleet enema before the examination. The subjects were evaluated in the left lateral decubitus position. A Peritron precision perineometer 9300AV (Cardio Design Pty Ltd, Oakleigh, Victoria, Australia) perineometer and 3010 (Cardio Design Pty Ltd, Oakleigh, Victoria, Australia) type anal sensor were used.

The anal probe was 80 mm in length and was produced to have a pressure-sensitive part of 30 mm in the middle. The average figure for serial measurements of 1 minute anal channel resting pressure of the subjects was recorded. The subjects were then told to contract the anal sensor as powerfully as they could and to hold it in contraction. This procedure was repeated 3 times with 10 second intervals and the data were recorded. The values with the most successful contraction were determined to be the manometric values of the subject^(13)^. Maximum contraction pressure values were taken into consideration in the evaluation of the subjects’ external anal sphincter (EAS) contractions. The pressure unit was taken in cm H_2_0 values in measurements conducted with the perineometer.

### Endoanal ultrasonography

The anal endosonography procedure was performed at the imaging laboratory of our hospital’s gastroenterology clinic. Imaging was conducted using a Fujinon ITD-01 EUS and a P2612M model flexible radial ultrasonic sonoprobe with 12 MHz frequency. All endoanal ultrasonography (EAUSG) procedures were performed by two physicians experienced in gastroenterology and proctology. The participants’ information about their mode of delivery was not shared with the physician performing EAUSG. The locations of all defects [OASIS and/or internal anal sphincter (IAS)] were located.

### Statistical Analysis

The mean, standard deviation, lowest, highest median, frequency, and percentage rates were used in the descriptive statistics of the collected data. The distribution of variables was measured using the Kolmogorov-Smirnov test. The Mann-Whitney U test was used for the analysis of quantitative data, and the chi-square test was used for the analysis of qualitative data. The effect level was investigated by univariate and multivariate logistic regression. SPSS 22.0 was used for all analyses.

## RESULTS

The demographic data of the premenopausal (group 1 + group 3) and postmenopausal (group 2 + group 4) groups are shown in Table 2. The episiotomies in vaginal deliveries were all mediolateral. There were no statistical differences between the groups regarding the participants’ body mass index (BMI), high birth weight, and instrument use (p>0.51).

The mean age and the number of deliveries of the patients in group 1 were similar to those of group 3. The endoanal ultrasonography imaging revealed that 20 (40%) and 2 (4%) patients in groups 1 and 3 had occult anal sphincter defects, respectively. Although sphincter defects were mostly seen in the EAS (24%) in group 1, only 2 patients were detected as having IAS defects in group 3 (Table 3). Anorectal manometric measurements showed that the maximum extrusion pressure, mean extrusion duration, and mean extrusion pressure values of group 1 were significantly lower than group 3 [odds ratio (OR): 1.02, 95% confidence interval (CI): 1.01-1.039; OR: 1.04, 95% CI: 1.02-1.05; OR: 1.03, 95% CI: 1.02-1.05; p<0.01, respectively). There was no significant difference (p>0.05) between group 1 and group 3 regarding WIS and the mean resting pressure values (Table 4).

There was no difference between group 2 and group 4 with regards to the mean age of the patients and the number of deliveries. Twenty-two (44%) patients in group 2 were detected to have occult anal sphincter defects according to endoanal ultrasonography data, and 2 (4%) patients in group 4 had sphincter defects (p<0.001). The most common sphincter defect seen in group 2 was EAS (24%). Anorectal manometric measurements showed that the maximum extrusion pressure, mean extrusion duration, mean extrusion pressure values of group 2 were significantly lower than group 4 (1.025 OR, 95% CI:0,46-0,78; p<0.001). There was, however, no significant difference between the groups regarding the mean resting pressure. The Wexner score of group 2 was significantly higher than group 4 (0.64 OR, 95% CI:[0.48-0.86]; p=0.003). Although 8 (16%) of patients had distinctive FI and 14 (28%) had incontinence symptoms in group 2, 10 (20%) patients had started to have fecal complaints in group 4 (Table 5).

**Figure 1:**
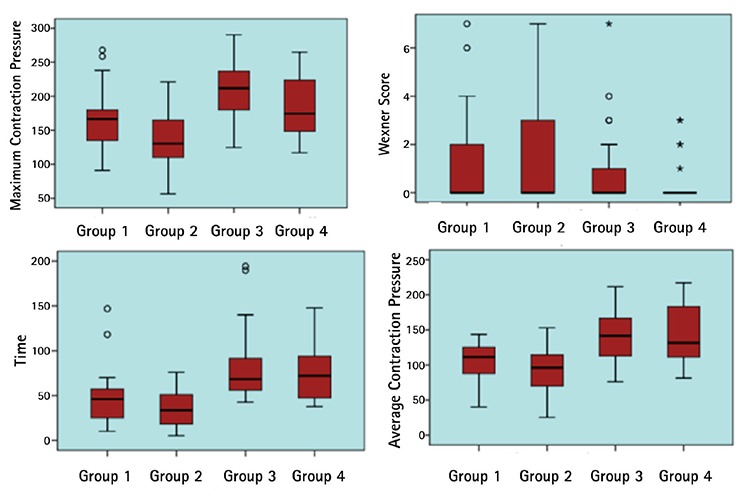
The distribution of maximum contrusion pressure, Wexner incontinence scale, mean contraction duration, and mean contraction pressure values of the participants according to the groups

When the patients who had had vaginal delivery were compared with regards to their menopausal status, there were no statistical differences between the groups according to EAUSG data with regards to the existence of sphincter defects and the location of defects (p=0.68). Anorectal manometric measurements showed that the maximum extrusion pressure, mean extrusion duration, mean extrusion pressure values of group 2 were significantly lower than group 1 (p<0.05). The Wexner scores of the groups were not significantly different (Table 6). Although no obvious FI was observed in group 1, 7 (14%) patients in group 2 had obvious FI. Figure 1 graphically demonstrates maximum contrusion pressures, WIS, mean contrusion durations, and mean contrusion pressure values of all groups.

## DISCUSSION

One of the most significant causes of FI in women is vaginal deliveries that result in anal sphincter injuries. Although OASIS is seen in 0.5-9% of all deliveries, it has been reported that 35-44% of women who had vaginal deliveries also had occult anal sphincter injuries when EAUSG began to be used by physicians^(6)^. However, FI symptoms are observed in only 20% of these women. Therefore, the natural history and significance of occult anal sphincter injuries still proves to be controversial^(14)^. In our study, we investigated the effects of occult anal sphincter injuries formed after vaginal deliveries on FI seen in the postmenopausal stage. Factors such as the number of deliveries, which increase the risk of FI after vaginal delivery, use of vacuum and forceps during delivery, BMI, existence and type of episiotomy, and birthweight were regarded as similar to those of the co-control groups. The premenopausal and postmenopausal anorectal manometric measurement data of women with vaginal deliveries were worse than women with c-sections (Table 6). During the course of our study, we observed that anal sphincter functions in post-vaginal delivery life were negatively affected regardless of the existence of FI symptoms. Furthermore, we ascertained that c-section had a protective effect both on OASIS and anal sphincter functions. Similarly, Hannah et al.^(15)^ also reported that c-section proved to be protective for postpartum FI symptoms.

When we compared the results of the premenopausal groups, we saw that 20 (40%) among the patients with vaginal delivery had occult anal sphincter injuries. When patients with vaginal deliveries were compared with the control group, there was no difference between FI symptoms and Wexner incontinence scores, although the former’s mean maximum extrusion pressure was lower (p>0.05). Studies in literature have reported that as EAUSG went into effect in medical practice, the rate of sphincter injuries reached 35% in primiparous women and 40% in multiparous women^(16)^. Moreover, FI symptoms in these patients were observed less than sphincter injuries (13% and 23%, respectively)^(17)^. Thus, some sphincter injuries formed during vaginal delivery do not result in FI. It is highly likely that remnant sphincter tissue is exposed to hypertrophy and enables the continuation of continence by increasing the amount of collagen in spite of the sphincter injury.

When the postmenopausal groups were compared, 44%(22) of the vaginal delivery group had sphincter injuries. When the vaginal delivery group was compared with the control group, however, it was ascertained that the former had both worse manometric measurement results and significantly higher WIS (p=0.03; Table 5). Based on these data, it can be suggested that anal sphincter injuries that formed after vaginal delivery in the premenopausal stage remained occult but they proved to be a factor, which give way to an increase in FI symptoms in the postmenopausal stage. In contradiction to our results, Mous et al.^(18)^ stated that the increase in FIs in the postmenopausal stage was related to postmenopausal disorders seen in the pelvic floor rather than sphincter injuries formed during vaginal delivery. With increasing age, and especially during the postmenopausal stage when estrogen in the body decreases, type 1 collagen tissue, which has thicker and stronger fibers, is replaced by type 3 collagen tissue, which has thinner, weaker, and isolated fibers. Moreover, IAS sclerosis develops with increasing age and atrophic changes take place in the EAS and pelvic floor muscles with the decrease in estrogen as menopause begins^(19)^. Furthermore, vaginal deliveries cause the pelvic diaphragm to move downward and give way to weakness in the pelvic floor^(20)^. When all these mechanisms are taken into consideration, it is clear that bodily changes in the postmenopausal stage increase FI. The results of our study revealed that patients with vaginal deliveries had worse results in all anorectal manometric measurements and higher WIS compared with those in the co-control groups when we compared the results of postmenopausal patients with vaginal deliveries and c-sections. Therefore, we believe that occult anal sphincter injuries become symptomatic with pelvic floor disorders formed in the postmenopausal stage, and they bring about a further increase in the incidence of FI. Our results are in parallel with former studies; however, two previous studies that compared more than 15-year follow-up results of patients with FI with or without anal sphincter injury reported contradictory results^(21,22)^. Nygaard et al.^(21)^ found no significant difference pertaining to FI between women with c-section delivery and those who had OASIS during normal delivery, and Faltin et al.^(22)^ conducted a study with similar groups and the authors reported that OASIS had little contribution to FI. The results of our study revealed that women with vaginal deliveries had a higher rate of anal sphincter injury and had higher WIS and FI symptoms in the postmenopausal stage compared with women with c-section deliveries in the same stage. Previous studies in the literature stated that FI symptoms increased depending on many factors in the postmenopausal stage^(20)^. The results of our study demonstrated that occult sphincter injury proved to be a significant factor that exacerbated these symptoms.

### Study Limitations

One of the limitations of our study is that we did not conduct research on pudendal nerve damage. There is, however, controversy over the effect of pudendal nerve damage formed during vaginal delivery on FI formation^(23)^. In a study by Sultan et al.^(6)^ the authors found that 16% of primiparous women and 15% of multiparous women had long-term pudendal nerve terminal motor latency (PNTML) 6 weeks after vaginal delivery, but there was no relationship between PNTML change and the development of FI symptoms. Further, abnormal PNTML prolongation had a statistically significant relationship with anal sphincter injuries. The results of another study showed that only one third of prolonged PNTMLs 6 months after delivery remained pathological^(24)^. Thus, we neglected to investigate the effects of pudendal nerve damage on FI in our study.

The other limitation of our study is that the participants in the pre- and postmenopausal groups were composed of different individuals. Pre- and postmenopausal data of participants in the same group could have rendered this study more significant. Instead, we selected participants with FI predisposing factors such as age, number of deliveries, BMI, birthweight, and instrument use to ensure similarity between the groups. This method of selection, in turn, contributed to the reliability of our study.

## CONCLUSION

Vaginal deliveries prove to be one of the most significant causes that increase the rate of anal sphincter injuries. Anal sphincter injury formed subsequent to vaginal delivery can be an important factor which gives way to an increase in the incidence of postmenopausal FIs and the formation of FI symptoms in women.

## Figures and Tables

**Table 1:**
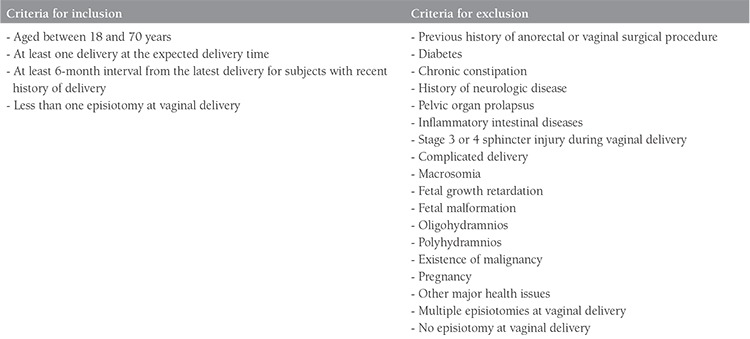
The study inclusion and exclusion criteria

**Table 2:**
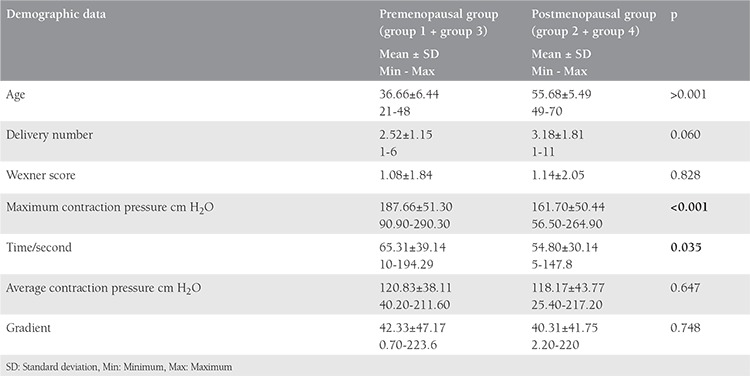
Demographic data of the participants according to premenopausal and postmenopausal stages

**Table 3:**
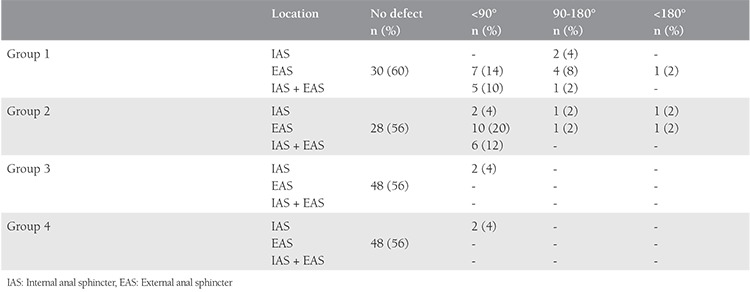
Endoanal ultrasound data of participants that location and size of defect at all groups

**Table 4:**
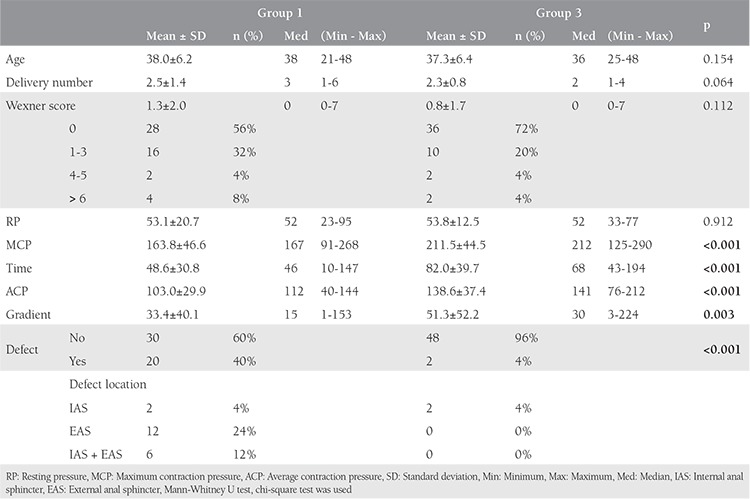
The comparison of demographic data, endoanal ultrasonography, and manometric measurement results of groups 1 and 3

**Table 5:**
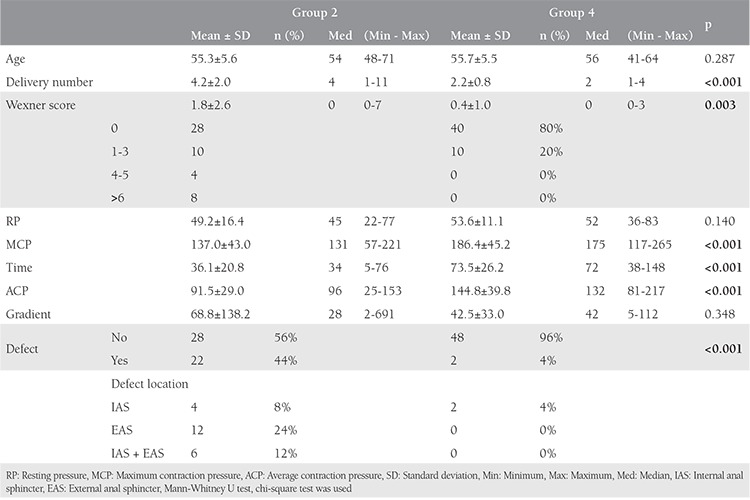
The comparison of demographic data, endoanal ultrasonography, and manometric measurement results of groups 2 and 4

**Table 6:**
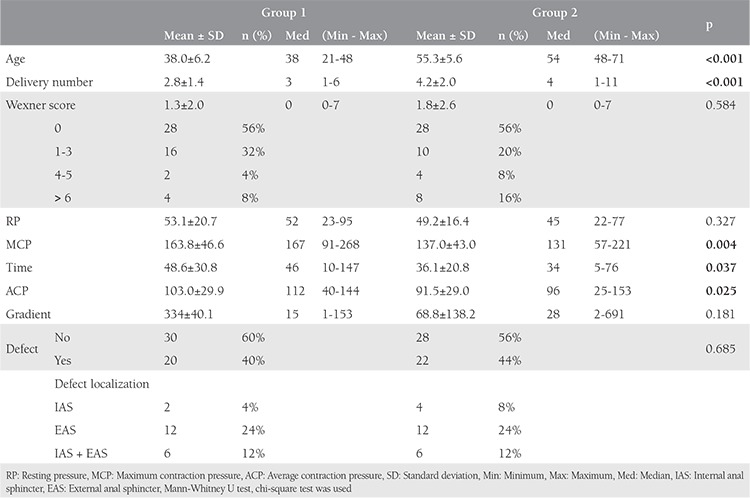
Comparison of vaginal delivery groups’ demographic, endoanal ultrasonography, and anorectal manometric data according to menopausal status
